# Gut microbiota composition as a candidate risk factor for dimethyl fumarate-induced lymphopenia in multiple sclerosis

**DOI:** 10.1080/19490976.2022.2147055

**Published:** 2022-11-18

**Authors:** Martin Diebold, Marco Meola, Srinithi Purushothaman, Lena K Siewert, Elisabeth Pössnecker, Tim Roloff, Raija LP Lindberg, Jens Kuhle, Ludwig Kappos, Tobias Derfuss, Adrian Egli, Anne-Katrin Pröbstel

**Affiliations:** a Departments of Neurology, Biomedicine and Clinical Research & Research Center for Clinical Neuroimmunology and Neuroscience Basel (RC2NB), University Hospital and University of Basel, Basel, Switzerland; b Institute of Neuropathology, Neurocenter, University Hospital Freiburg, University of Freiburg, Freiburg, Germany; c Division of Clinical Bacteriology and Mycology, University Hospital Basel, Basel, Switzerland; d Applied Microbiology Research, Department of Biomedicine, University of Basel, Basel, Switzerland; e Institute for Medical Microbiology, University of Zurich, Zurich, Switzerland; f Swiss Institute of Bioinformatics, Basel, Switzerland

**Keywords:** Multiple sclerosis, microbiome, dimethyl fumarate, lymphopenia, metabolomics, therapeutic biomarker

## Abstract

Mounting evidence points towards a pivotal role of gut microbiota in multiple sclerosis (MS) pathophysiology. Yet, whether disease-modifying treatments alter microbiota composition and whether microbiota shape treatment response and side-effects remain unclear. In this prospective observational pilot study, we assessed the effect of dimethyl fumarate (DMF) on gut microbiota and on host/microbial metabolomics in a cohort of 20 MS patients. Combining state-of-the-art microbial sequencing, metabolome mass spectrometry, and computational analysis, we identified longitudinal changes in gut microbiota composition under DMF-treatment and an increase in citric acid cycle metabolites. Notably, DMF-induced lymphopenia, a clinically relevant safety concern, was correlated with distinct baseline microbiome signatures in MS patients. We identified gastrointestinal microbiota as a key therapeutic target for metabolic properties of DMF. By characterizing gut microbial composition as a candidate risk factor for DMF-induced lymphopenia, we provide novel insights into the role of microbiota in mediating clinical side-effects.

## Introduction

While studying gut microbiota is a promising approach to better understand pathophysiology and open novel therapeutic avenues in multiple sclerosis (MS), we lack fundamental knowledge about how disease-modifying therapies (DMTs) affect gut microbiota composition and metabolism. Further, it remains unclear whether distinct microbial taxa shape treatment response and drug-induced side-effects^1^.

In this context, recent publications^2–6^ set a spotlight on dimethyl fumarate (DMF), a frequently administered DMT. DMF is a *bona fide* immunometabolic compound with metabolic properties on the mitochondrial respiration of activated T cells^5^ and restrictive effects on glycolysis in lymphocytes.^2,6^ Yet, it is likely that the effects of this empirically developed, untargeted, small molecule reach beyond T cells, potentially affecting other metabolically active human and bacterial cells. Notably, DMF, a citrate cycle derivative, is rapidly metabolized within the gastrointestinal tract and causes self-limited gastrointestinal side-effects in many patients.^7^ Indeed, cross-sectional observations in MS patients indicated that DMF may affect not only immune cells but also the gastrointestinal microbiota^8–10^, and thereby potentially reduce the release of direct neurotoxic compounds.^3^ However, knowledge regarding longitudinal alterations of the gut microbiota under DMF and their metabolic consequences is limited. We used the setting of an established prospective observational study,^6^ to explore the effect of DMF on the metabolic profile of the host and on the gut microbiome. Further, we addressed whether distinct microbial signatures can be linked to treatment response and DMF-induced side-effects.

## Results

**Figure 1.**
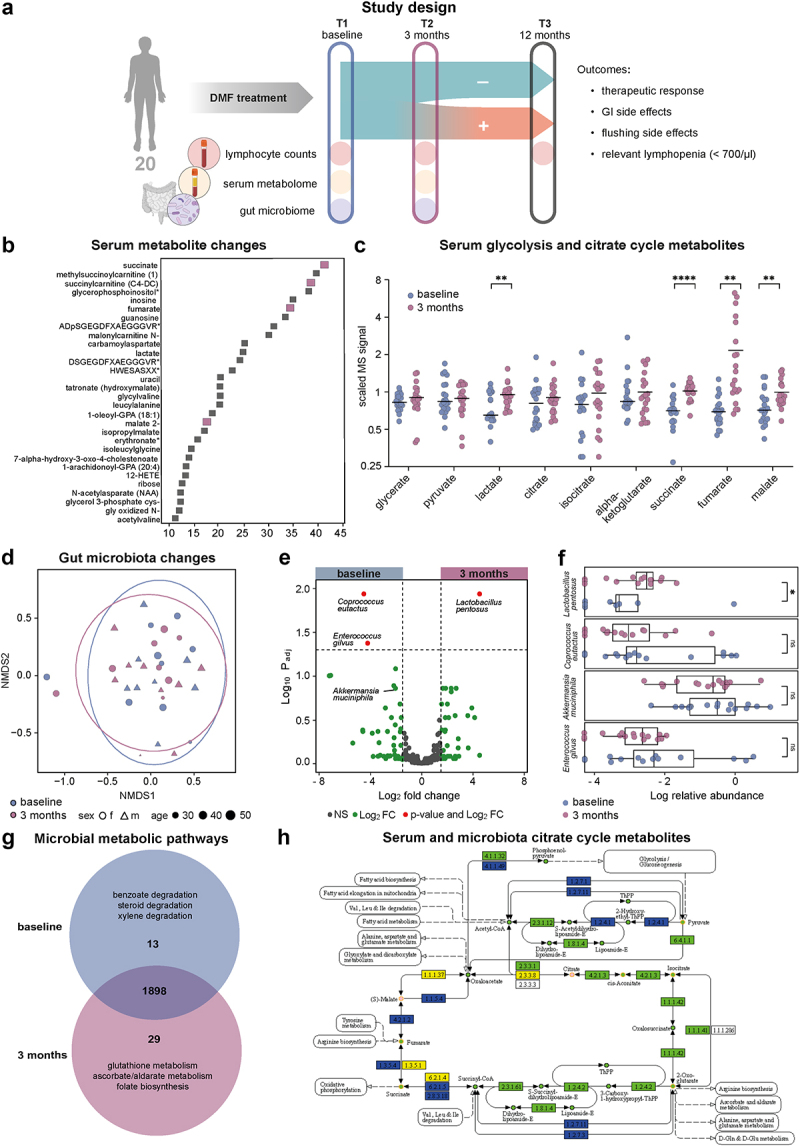
**DMF treatment specifically recomposes the gut microbiota**. (a) In this prospective observational study, serum metabolome, gastrointestinal microbiota as well as clinical and paraclinical parameters were assessed in 20 individuals during the first year of oral DMF treatment to delineate markers of clinical outcomes. (b) Random Forest analysis of longitudinal changes within all 805 identified serum metabolites. Higher mean decrease accuracy suggest more relevant longitudinal changes (top 30 metabolites shown), citrate cycle metabolites are marked in purple. (c) Glycolysis and citrate cycle metabolites, as measured by mass spectrometry, compared between baseline and 3 months timepoints. (d) Non-metric multidimensional representation (NMDS) of the gastrointestinal microbiota composition of baseline (blue) and 3 months (purple) samples (stress: 0.17). Circles represent confidence interval of 95%. (e) Volcano plot of 3 months/baseline microbial species, significantly (p_adj_<0.05) overrepresented species at baseline (left) or 3 months (right) illustrated in red. (f) Box plots of log10-transformed normalized relative abundances of the four most affected (according to volcano plot, 1E) microbial species at baseline (blue) and 3 months (purple). (g) Venn diagram from AMON analysis for baseline vs. 3 months for metabolic pathways of the gastrointestinal microbiota. (h) Visualization of AMON analysis of putative origin of metabolites (circles) and enzymatic reactions (rectangles) of the citrate cycle, with dark blue indicating exclusive bacterial origin, yellow indicating exclusive human origin, and orange indicating compounds detected in serum. Green color labels compounds of either human or bacterial origin without certain attribution.

We enrolled 20 patients with relapsing-remitting MS (RRMS), a mean age of 42 y (range 23–59), and marginal female preponderance (55%) (Table 1). Exploring the imprint of DMF on the host serum metabolome and gut microbial metabolome in a longitudinal setup, we assessed early changes in patients’ metabolomes and microbiomes and compared them to outcomes during 12 months under DMF therapy (Figure 1a).

**Table 1.** Clinical data of the study cohort (baseline characteristics and outcomes of each individual of the study cohort).

Baseline characteristics									Outcomes			
Patient ID*	Age (a)	Sex	Disease course	Disease duration (a)	Previous disease activity (relapses/2a)	Previous DMT	Delay from previous DMT (d)	EDSS at baseline	Disease activity (1: MRI, 2: MRI + relapse)	Lymphopenia (<700/µl in first year)	GI symptoms	Flushing
1	36	m	RRMS	0.3	0	None	-	1.0	0	0	0	1
2	38	m	RRMS	0.2	2	None	-	2.0	0	0	0	0
3	45	f	RRMS	3.8	0	Daclizumab	27	2.0	0	1	0	1
4	59	f	RRMS	3.4	0	Fingolimod	104	3.5	0	1	0	0
5	54	f	RRMS	8.1	0	Glatirameracetate	12	3.0	0	0	1	1
6	43	m	RRMS	2.3	0	Teriflunomide	53	2.0	0	1	1	1
7	50	f	RRMS	11.7	0	Interferon beta	546	2.5	0	1	0	1
9	57	f	RRMS	0.9	2	None	-	3.0	0	1	0	0
10	40	f	RRMS	0.3	1	None	-	1.5	0	1	0	0
13	32	m	RRMS	0.4	1	None	-	1.0	2	0	0	0
14	23	m	RRMS	0.2	1	None	-	2.0	1	0	0	1
15	42	m	RRMS	0.2	2	None	-	3.0	0	0	1	1
16	37	f	RRMS	7.4	1	Teriflunomide	74	1.5	0	1	1	1
17	44	m	RRMS	10.2	0	Fingolimod	115	4.0	0	0	0	0
19	30	m	RRMS	0.1	2	None	-	2.0	0	0	0	0
21	49	f	RRMS	0.1	1	Glatirameracetate	16	1.5	2	1	0	0
25	34	m	RRMS	1.0	0	Glatirameracetate	17	0.0	1	0	0	0
27	27	f	RRMS	6.5	4	Teriflunomide	62	3.0	0	0	0	1
29	50	f	RRMS	10.6	0	Glatirameracetate	5	1.0	1	1	0	0
33	41	f	RRMS	0.1	1	None	-	2.5	0	0	0	0

### DMF alters host and microbiota metabolome

Assessing patient sera, a total of 805 compounds of known identity were recognized by ultrahigh-performance liquid chromatography-tandem mass spectroscopy (UPLC-MS/MS), of which 31 statistically significantly increased (n = 21) or decreased (n = 10) during the first 3 months of treatment (corrected for False Discovery Rate, q < 0.05). Additionally, we conducted a random forest (RF) analysis comparing baseline and three-month groups to rank these effects (Figure 1b). By both approaches, several of the top 30 biochemicals predicting group separation were linked to essential energy metabolism, specifically containing increased intermediates of the citrate cycle. The most significant increases were identified for fumarate (2.3-fold, q = 0.008) and its neighboring metabolites succinate (1.5-fold, q = 0.006) and malate (1.3-fold, q = 0.016) (Figure 1c), in serum.

Next, we assessed longitudinal changes in the gut microbiome by amplicon sequencing (rarefaction curve **e**Figure 1a). While no significant changes in the alpha diversity (**e**Figure 1b) and no separate grouping in a non-metric multidimensional scaling (NMDS) representation were observed regarding DMF treatment (Figure 1d**, e**Figure S1c) or previous treatments (**e**Figure S1d), we identified significantly decreased (n = 2) and increased (n = 1) abundance of a few bacterial species under DMF treatment (Figure 1e). The group of decreased bacteria prominently included *Coprococcus eutactus* (P_adj_ = 0.012), *Enterococcus gilvus* (P_adj_ = 0.042), and – though not significant – *Akkermansia muciniphilia* (P_adj_ = 0.138). *Lactobacillus pentosus* (P_adj_ = 0.012) was significantly increased upon treatment (Figure 1e-f**, eTables S1–2**). To compare the metabolic alterations on the host (patient) and the microbiota (**e**Figure S1e-h), we used PICRUSt2 for imputing microbial metabolomics from 16S sequencing data. In the gut, 13 metabolites were identified uniquely before treatment onset and 29 metabolites only after 3 months (Figure 1g**, eTable S3**). Complementing the serum metabolite analysis, an increased abundance of citrate cycle intermediates was observed in the microbiome profile under DMF treatment. Under treatment, we furthermore detected compound C05422, involved in antioxidative ascorbate, aldarate, and glutathione metabolism (Figure 1h).

### DMF-induced lymphopenia associates with distinct baseline microbiome signatures

**Figure 2.**
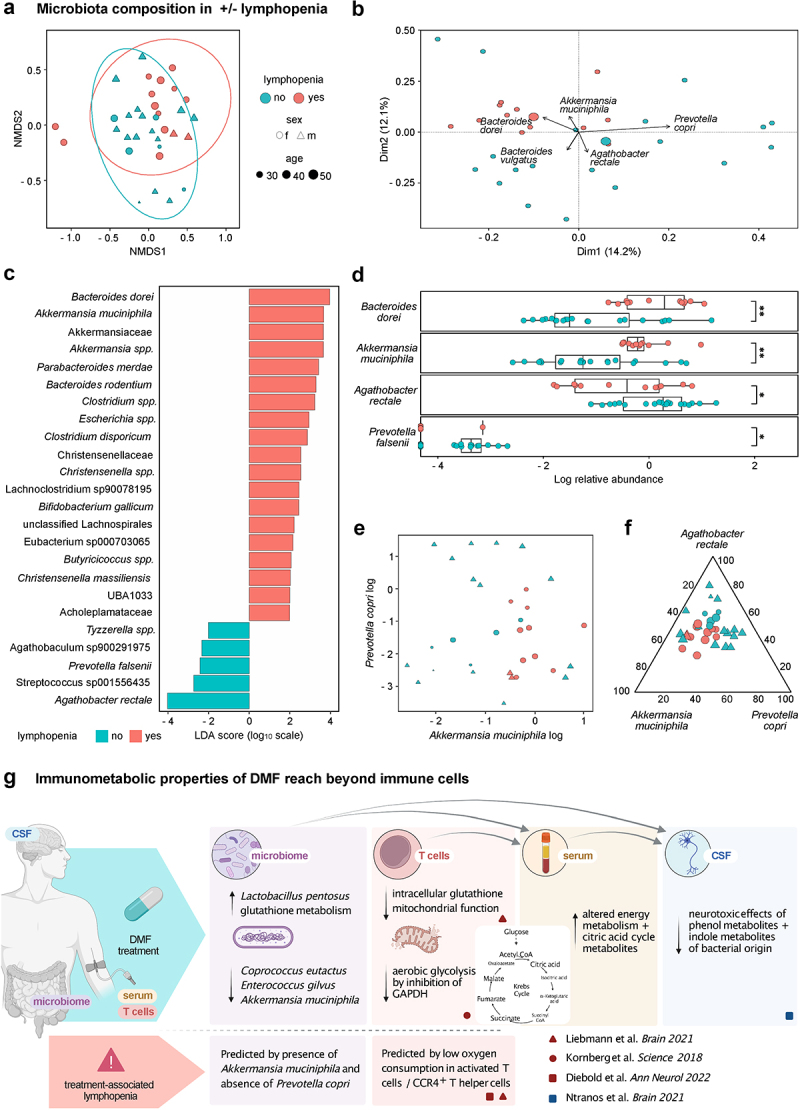
**Microbiota composition predisposes to DMF-associated lymphopenia**. (a) Non-metric multidimensional representation (NMDS) of the gastrointestinal microbiota composition of samples from individuals with (red) or without (turquoise) subsequent DMF-associated lymphopenia. Circles represent confidence interval of 95% (stress: 0.17). (b) Principal component analysis of the gastrointestinal microbiota composition of samples from individuals with (red) or without (turquoise) subsequent DMF-associated lymphopenia with Euclidean vectors representing the effects of main microbiota, excluding one individual with dominant streptococcal effect (b–f). (c) LEfSe analysis of dominant gastrointestinal microbiota of individuals with (right) and without (left) subsequent DMF-associated lymphopenia. Colored bars indicate taxa highlighted in adjunct figures. (d) Box plots of log10-transformed normalized relative abundances of four most affected (according to principal component analysis, 2B, and LEfSe analysis, 2C) microbial species in samples with (red) or without (turquoise) subsequent DMF-associated lymphopenia. (e) Bi-axial dot plot of log10-transformed relative abundances of *Akkermansia muciniphila* and *Prevotella copri*, color code indicating samples with (red) or without (turquoise) subsequent DMF-associated lymphopenia. (f) Ternary dot plot of the log_10_-transformed and subsequently normalized abundances of *A. muciniphila, Agathobacter rectale*, and *P. copri* only, color code indicating samples with (red) or without (turquoise) subsequent DMF-associated lymphopenia. (g) Graphical summary of findings from this study and the recent literature.

We next assessed correlations between clinical outcomes and microbiota composition. Notably, patients with or without subsequent treatment-associated lymphopenia clustered based on their microbiome composition (Figure 2a). By contrast, we observed no altered patterns in the NMDS representation for disease activity, gastrointestinal, or flushing side effects (**e**Figure S2a-c). With the exception of patient 16 (microbiota characterized by high abundances of *Streptococci*), all samples of the lymphopenia group showed a similar taxonomic pattern already at baseline. To assess this lymphopenia-associated microbiome composition, we identified the most relevant single species for development of lymphopenia (Figure 2b-c, **e**Figure S2d-e, metabolic features **e**figure S2f-i, excluding patient 16 due to its outlier status, **eTables S4–6**). In two independent analyses of our dataset, we identified a group of bacteria composed of *A. muciniphila, Bacteroides dorei, Agathobacter rectale, Prevotella copri*, and *P. falseni* (by LEfSe Figure 2c-d, by Euclidean vector assessment of a principal component analysis Figure 2b and **e**Figure S2d). Notably, these species discriminated between patients with and without subsequent lymphopenia before treatment (**e**Figure S2e). We observed a constant presence of *A. muciniphilia* and absence of *P. copri* with the occurrence of lymphopenia. Conversely, subjects with no lymphopenia were characterized by the presence or absence of both *A. muciniphilia* and *P. copri* or the absence of *A. muciniphila* in presence of *P. copri* (Figure 2e). Sensitivity and specificity of lymphopenia prediction as side effects based solely on these two species was 0.85. To determine a third species potentially increasing the discriminatory power between patients with and without subsequent lymphopenia, we tested several combinations of ternary plots composed of *A. muciniphila, P. copri*, and a third species candidate – such as *A. rectale –* highlighted in the previous multivariate analyses (Figure 2f and **e**Figure S2j).

## Discussion

In this study, we aimed to assess differences in the metabolomes of patients and their microbiota related to DMF treatment and identified bidirectional treatment-microbiota associations. While the treatment *per se* influenced the microbiota composition, a specific constellation of certain commensal taxa served as a candidate predictor for the occurrence of lymphopenia under treatment.

Our findings demonstrate a specific rearrangement of MS-associated taxa^11^ upon DMF treatment with a decrease in MS-associated proinflammatory taxa, such as *A. muciniphilia* and *C. eutactus*,^1,12,13^ and an increase in allegedly beneficial, anti-inflammatory species, like *L. pentosus*,^10,14^ substantiating previous cross-sectional data.^8–10^ Detailing the metabolic properties of DMF,^2,5,6^ we demonstrated increasing late citrate cycle intermediates not only in the host’s serum (where they had previously been hypothesized) but also in the microbial metabolism. Whereas these beneficial immunomodulatory effects in two compartments may be explained by simultaneous metabolic changes in bacteria and blood under DMF, an alternative interpretation would identify the changing gastrointestinal microbiota composition as the reason for a cascade of effects, including lymphocyte alterations and a loss of neurotoxic bacterial metabolites observed in the serum and cerebrospinal fluid of patients^3^ (Figure 2g). The latter hypothesis may be supported by the short half-life of fumaric acid esters with rapid degradation in the citrate cycle. The gut microbiota are therefore privileged over circulating immune cells by their early, metabolically relevant contact with DMF. In light of the very limited understanding of the direct effects of fumaric acid esters on specific bacteria,^15,16^ further research will be needed to dissect metabolic properties of DMF on microbiota – and their effects on host immune cells.

Further, our observation that baseline microbiota composition may be a critical mediator of lymphopenia, as a side effect of DMF, suggests that the drug-microbiome interplay reaches beyond these therapeutic effects. Lymphopenia represents the main safety concern of DMF treatment due to its association with rare cases of progressive multifocal leukoencephalopathy.^17^ While it is known that patients with relevant lymphopenia are older and have lower baseline lymphocyte counts – specifically of CCR4^+^ T helper cells –^4,18^ more refined biomarkers for risk stratification of this severe side effect are utmost needed to tailor personalized treatment recommendations. Notably, we here observed an association between the baseline presence of *A. muciniphilia* and absence of *P. copri* with the development of lymphopenia during treatment. In turn, lymphopenia was absent in patients with simultaneous appearance of both *A. muciniphila* and *P. copri*, indicating a possible counteracting effect by the presence of *P. copri*. Of interest, these taxa were previously described in association with MS and other autoimmune disorders. Namely, *A. muciniphilia* and *B. dorei*^1,19^ – both overrepresented in lymphopenia patients – and *P. copri* were shown to be reduced in MS patients^12^ but increase upon DMT.^11^ While we cannot exclude that this small exploratory pilot study may have been affected by unrecorded dietary effects or under-powered to identify associations for rare events – including an association of gut microbiota with disease activity^20^ – observations from this first longitudinal microbiome analysis under DMF provide pivotal evidence for a role of microbiota in the development of lymphopenia. We therefore believe that larger studies are warranted to evaluate the feasibility of microbiome testing for the prediction of DMF-associated lymphopenia in MS patients with implications for the assessment of microbiota in mediating clinically relevant side-effects under other DMTs.

## Patients and methods

We prospectively collected stool and blood samples from 20 patients with RRMS intending to start DMF treatment, as an extension of the project previously published by Diebold et al. in 2018.^6^ Sampling for assessment of microbiome or metabolome predictors was scheduled at two early time-points: before first intake (baseline) and after 3 months of DMF treatment. Stool samples were collected using fecal collection kits (Epitope Diagnostics Inc., San Diego, CA) and stored at −80°C until further processing.

To cover a longer clinical and immunological follow-up, additional blood samples were collected after 12 months of DMF treatment. Blood samples from all three time points were assessed by automated flow cytometry for lymphocyte counts and major lymphocyte subpopulations (eTable 6). Detailed clinical information including survey of anamnestic and laboratory adverse events was collected at each time point. Assessed outcomes comprised adverse events (flushing and gastrointestinal side effects, relevant lymphopenia (<700/μl)) and disease activity (as judged by clinical relapse and MRI activity) within the first 12 months of treatment. Disease activity was assessed by recording clinical relapses and/or new lesions in T2-weighted cerebral magnetic resonance imaging (MRI) within 12 months after treatment started (Figure 1a). All participants gave written consent before enrollment. The study was conducted as a nested project within the Swiss Multiple Sclerosis Cohort Study and approved by the Ethics Committee for Northwest and Central Switzerland (EKNZ 201448/12).

### Serum metabolomics

Serum samples from the first 14 patients (available at the time point of analysis) within this cohort were characterized for metabolomics by UPLC-MS/MS, as described in.^6^ Each sample was analyzed using acidic positive ion conditions, basic negative ion optimized conditions, and negative ionization. Blinded data were processed and normalized by Metabolon Inc. (Durham, USA) for all assessed samples as described previously.^6^ Results were verified by paired analysis (for time points T1 and T2) for each observation and assessed using RF analysis on all measured metabolites.

### Amplicon sequencing

Stool samples of all 20 patients were assessed for their gut microbiome composition using the QIAseq 16S/ITS screening panel (QIAGEN), sequenced on a MiSeq (Illumina). In short, samples were thawed and total DNA (totDNA) from 250 mg stool was extracted with a QIAamp PowerFecal Pro DNA Kit (QIAGEN). Sequencing libraries were generated with the QIAseq 16S/ITS Screening Panels kit. For each sample, 1 ng of totDNA was amplified with each of the three Screening Panel Pools and subsequently combined and indexed. The indexed libraries were pooled equimolarly, and 15pM were loaded and sequenced PE 276 on a MiSeq v3 600-cycle kit (Illumina).

### Bioinformatic & statistical analysis

The bioinformatic analysis was performed on R (R core team) using the phyloseq package (v.1.38.0).^21^ Operational taxonomic unit (OTU) abundances were calculated in the CLC microbiome analysis tool. Taxonomic annotation of the OTU reference sequences was done with the GTDB R89 database with full 16S^22^ and IDTAXA algorithm^23^ with the DECIPHER package (v.2.22.0) in R (v4.1.2) (R Core Team 2020).

The obtained OTU abundance table, OTU reference sequences, taxonomic annotation, and metadata were imported into a phyloseq object for downstream statistical analyses with the phyloseq package (v.1.38.0)^21^ in R. Samples counts varied between 196166 and 846999 with a median count value of 359300. Out of the 8667 OTUs obtained after the bioinformatic analysis, 3176 were removed as singletons and present in <1% of the samples representing 0.073% of the overall abundances. A total of 5491 OTUs were used for downstream analyses representing 29 phyla, 55 classes, 119 orders, 194 families, 429 genera, and 914 species. All samples reached sufficient sequencing depth according to the rarefaction curve based on the detected species.

Alpha diversity indices based on species composition (richness, Shannon, Simpson, inverse Simpson, and chao1) were assessed using the phyloseq package in R. NMDS was analyzed in R with the phyloseq package using compositional species table and Bray-Curtis algorithm. Principal component analysis (PCA) plots with contribution arrows as explanatory variables were calculated in R with the factoextra package (v.1.0.7) using Hellinger transformed compositional data of species composition. The Euclidean vectors were calculated using the function prcomp() of the package “stat” and fviz_pca_biplot() of the package “factoextra.” PCA with imputed metabolic compounds from PICRUSt2 was performed on Hellinger transformed abundance data.

Differential expression analysis of the bacterial species in baseline vs. 3 months and lymphopenia vs. no lymphopenia was performed with DESeq2 package (v.1.34.0)^24^ in R. Deseq function was run on a matrix not normalized species compositional data from phyloseq with the parameter test = “Wald” and fitType = “parametric”.

Boxplots were analyzed on normalized and log10 transformed relative abundance data of species composition. Samples were compared based on time point (baseline vs. 3 months) and side effect (no-lymphopenia vs. lymphopenia). Significance was assessed with the compare_mean function of the ggpubr package in R using method = ”wilkox.test” and p.adjust.method = ”hochberg.”

Ternary plots were generated using the ggtern package (v.3.3.5) in R. Species count abundance data were log10 transformed and the values of the three selected species normalized. The samples were plotted according to the ratio of the log10 abundance data of the three selected species.

Functional annotation for the microbiome was performed using PICRUSt2.^25^ The biom table was given as input to PICRUSt2. The entire pipeline was launched using PICRUSt2_pipeline.py command with default parameters.

Relative abundance OTU table and predicted pathway abundance tables are used as input for the LEfSe analysis. The OTUs and pathways that differ significantly between the time point groups were inferred from the analysis (http://huttenhower.sph.harvard.edu/galaxy) with default settings. The default cut-off score of 2 was used for logarithmic LDA score.

AMON was used to predict the origin of metabolites from host (human) and microbiome.^26^ Host metabolites with KEGG ids detected from the LC/MS and KEGG Orthologs, which are predicted for the microbiome from PICRUSt2, were given as input to AMON along with the complete flat files (reaction, ko, compound, and pathways) obtained from KEGG FTP (Academic license).^27–30^ AMON analysis is carried out for the baseline and treatment groups separately. Venn diagram method (Jvenn http://jvenn.toulouse.inra.fr/app/example.html) was used to identify metabolites, which were unique to the microbiome at baseline and 3 months under treatment.

## Supplementary Material

Supplemental Material

## Data Availability

Data will be made available in the EMBL-EBI repository [accession ID: PRJEB55040] upon publication of the manuscript.
