# Supplementary figures and images for: Gut microbiota composition as a candidate risk factor for dimethyl fumarate-induced lymphopenia in multiple sclerosis
Source: Gut Microbes. 2022 Nov 18;14(1):2147055. doi: 10.1080/19490976.2022.2147055 (PMC9677991; doi:10.1080/19490976.2022.2147055)

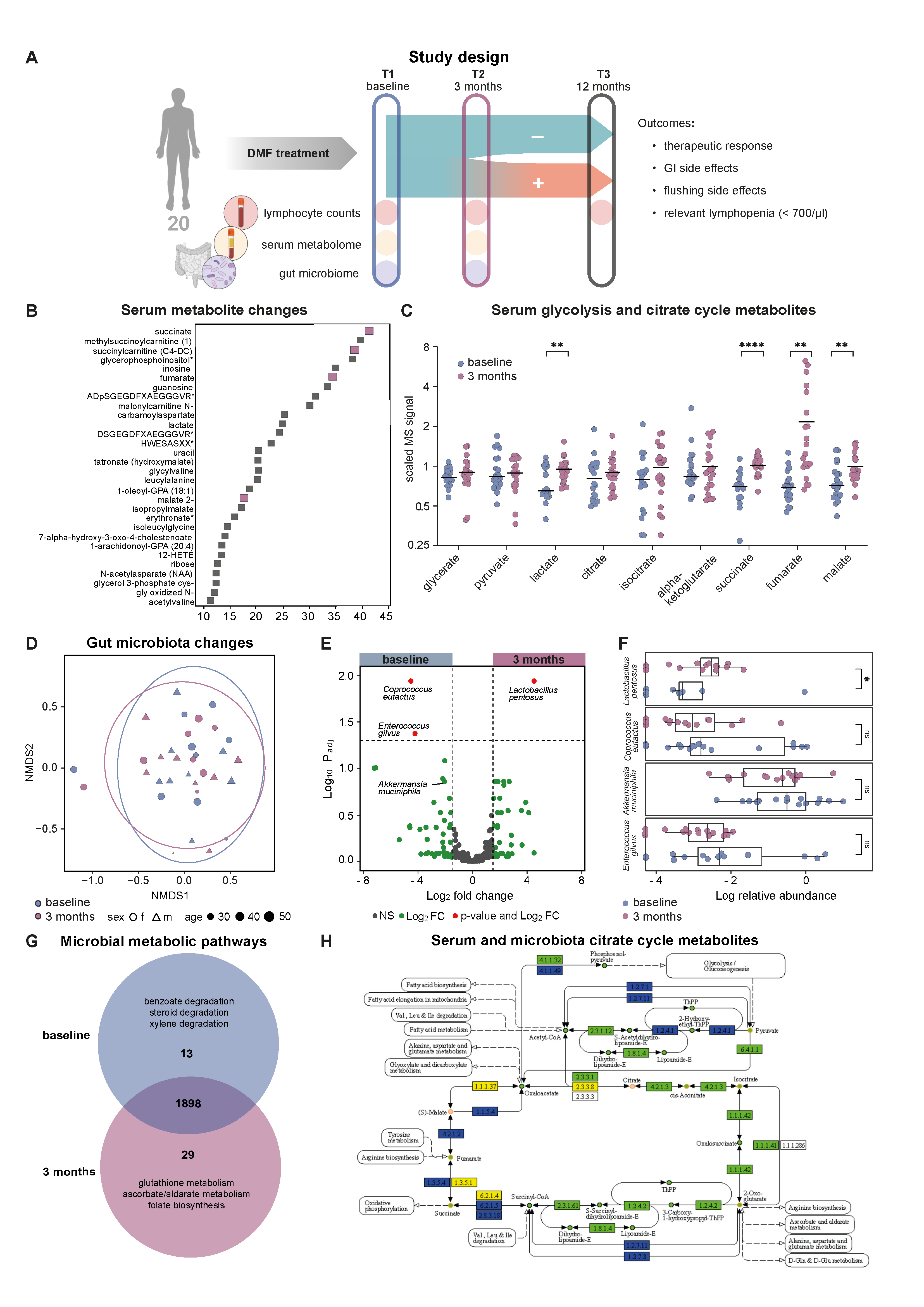

Supplement: Supplemental Material [file KGMI_A_2147055_SM2108.zip › eFigure1.tiff]

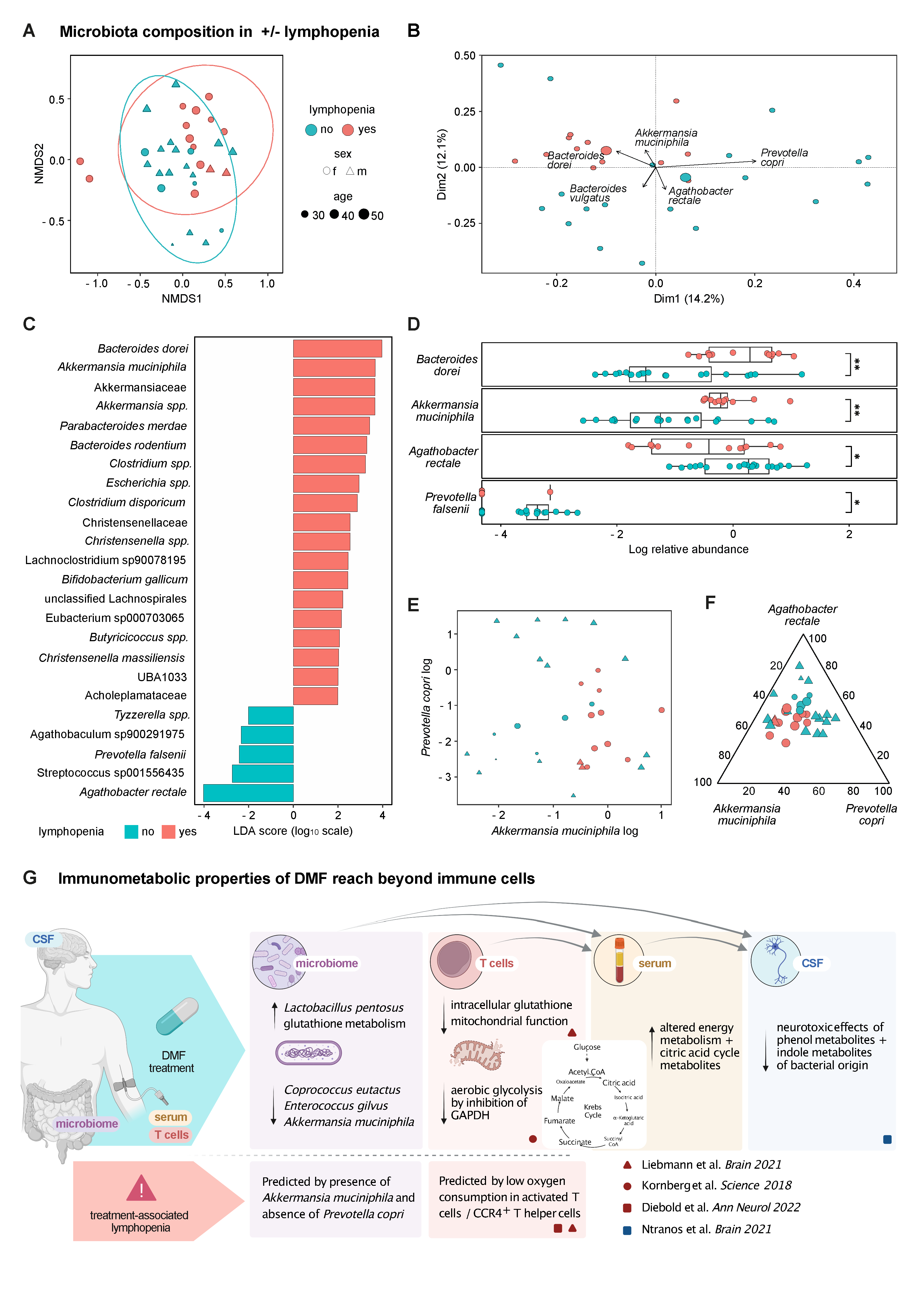

Supplement: Supplemental Material [file KGMI_A_2147055_SM2108.zip › eFigure2.tiff]
